# Incubation environment impacts the social cognition of adult lizards

**DOI:** 10.1098/rsos.170742

**Published:** 2017-11-22

**Authors:** Harry Siviter, D. Charles Deeming, M. F. T. van Giezen, Anna Wilkinson

**Affiliations:** 1 School of Life Sciences, University of Lincoln, Lincoln, LN6 7DL, UK; 2 School of Biological Sciences, Royal Holloway, University of London, Egham, Surrey, UK; 3 Faculty of Veterinary Medicine, Utrecht University, Utrecht, The Netherlands; 4 Wildlife Research Center, Kyoto University, 2-24 Tanaka-Sekiden-cho, Sakyo, Kyoto, 606-8203, Japan

**Keywords:** incubation, lizard, oviparous, social cognition, social learning, gaze following

## Abstract

Recent work exploring the relationship between early environmental conditions and cognition has shown that incubation environment can influence both brain anatomy and performance in simple operant tasks in young lizards. It is currently unknown how it impacts other, potentially more sophisticated, cognitive processes. Social-cognitive abilities, such as gaze following and social learning, are thought to be highly adaptive as they provide a short-cut to acquiring new information. Here, we investigated whether egg incubation temperature influenced two aspects of social cognition, gaze following and social learning in adult reptiles (*Pogona vitticeps*). Incubation temperature did not influence the gaze following ability of the bearded dragons; however, lizards incubated at colder temperatures were quicker at learning a social task and faster at completing that task. These results are the first to show that egg incubation temperature influences the social cognitive abilities of an oviparous reptile species and that it does so differentially depending on the task. Further, the results show that the effect of incubation environment was not ephemeral but lasted long into adulthood. It could thus have potential long-term effects on fitness.

## 1. Introduction

Environmental change is increasingly impacting habitats worldwide, creating novel challenges for the animals living there [1]. Genetic adaptation can be slow and, therefore, one of the first responses that an animal can make in the face of environmental change is behavioural [2]. Cognitive abilities are likely to play a major role in behavioural adaptation as they influence how an animal perceives, stores and uses information from the surrounding environment [3].

Reptiles are especially interesting in this context because of their dependence on behavioural thermoregulation [4] and their pattern of reproduction, which is reliant on environmental sources of heat for maintenance of embryonic development. Viable incubation temperatures for reptiles are wide-ranging and are known to impact upon many aspects of offspring phenotype, *inter alia* growth rate, sex determination and many aspects of behaviour (for a review see [5]). Recent work exploring the relationship between early environmental conditions and cognition has shown evidence that incubation temperature can influence brain anatomy [6] and performance in various learning tasks [7–9] in young Eastern three-lined skinks (*Bassiana duperreyi*). However, it is currently unknown whether incubation temperature impacts upon other aspects of cognition and the duration to which it might influence cognitive processes.

Social cognition encompasses all cognitive processes involved in acquiring knowledge from another individual and is typically studied in two categories, social intelligence and social learning [10]. One classic test of social intelligence, i.e. intelligence applied to the social world [11], is the use of gaze following, which refers to an animal's ability to follow the direction of another individual's gaze [12]. Such a skill is considered highly adaptive as it can alert the observer to essential information, such as the presence of a food source or predator. Two different modes of gaze following are typically observed. Firstly, gaze following into distant space, which is taxonomically widespread [12–15], and is likely to be controlled by a socially facilitated orienting response that can be modified through experience [13]. By contrast, geometric gaze following, which requires following gaze behind a visual barrier, is considered more complex as it entails an assessment of the difference in the visual perception between the cue-giver and the observer [14]. Thus, investigating two modes of gaze following will provide further insight into the level of complexity that egg incubation temperature may influence upon social cognition. Gaze following into the distance has been demonstrated in the red-footed tortoise [15] while geometric gaze following has never been investigated in reptiles and has only been observed in primates, corvids and canids [16–21].

Social learning, in its broadest sense, can be considered as ‘learning that is influenced by observations of, or interaction with, another animal (typically a conspecific) or its products’ [22]. Social learning is thought to be adaptive as it offers an individual a short-cut to novel information [23–24] such as potential resources [25] and can aid in learning novel foraging techniques [26]. Social learning is thought to be particularly advantageous when the costs of asocial learning are high [27], such as in areas of high predation or dwindling resources (e.g. [28]). Social learning is also positively correlated with asocial learning abilities [24] and it has been suggested that they are controlled by the same mechanisms [29–31]. Egg incubation temperature is known to influence associative learning in oviparous reptiles [6–9,32] and thus it is possible that these abilities could be extended to social learning abilities.

The long-term effects of egg incubation temperature on cognitive traits in oviparous reptiles are currently unknown. Typically, experiments use animals that are a few days or a few weeks old [6–9,32]. However, it remains unclear how long these differences last. In the only study to investigate this so far, we observed differences in the development of behavioural traits in bearded dragons (*Pogona vitticeps*) as a result of incubation temperature, with animals from the hot group initially appearing bolder than those in the cold group. However, these differences did not last into adulthood [33]. It is, therefore, essential to investigate the long-term impact of incubation environment on offspring phenotype.

Incubation temperature is known to influence the development of behavioural traits and the learning ability of oviparous reptiles [6–9,32,33], traits that are intrinsically linked to social cognition [3]. Thus, to begin to explore the association between incubation environment and social cognition, this study tested the impact that incubation environment has on gaze following into the distance, geometric gaze following and social learning abilities of adult bearded dragons.

## 2. Material and methods

Thirteen eggs were randomly assigned to two incubation conditions, the ‘hot group’ (*n* = 7) incubated at an average temperature of 30 ± 3°C, and the ‘cold group’ (*n* = 6) incubated at an average temperature of 27 ± 3°C (fully described in [33]). The eggs were incubated in multiple plastic boxes with a vermiculate substrate and kept moist. Bearded dragons do not have temperature-dependent sex determination when incubated at optimal temperatures [34] and hence, after incubation, we had an even spilt of sexes between incubation groups (hot group: four males and three females; cold group: three males, three females). Once hatched, the animals were housed in similar environments and maintained under standard conditions. Bearded dragons were social housed in heated vivariums (145 × 48 × 60 cm) with conspecifics from the same incubation temperature regime. The average temperature of the room was maintained at 29°C and all lizards received the same feeding regime. All bearded dragons had experimental experience [33] but had no previous experience with video experiments. The animals were at least one-year old at the time of testing for both experiments (see below) and were all considered to be sexually mature.

### 2.1. Gaze following

**Figure 1.**
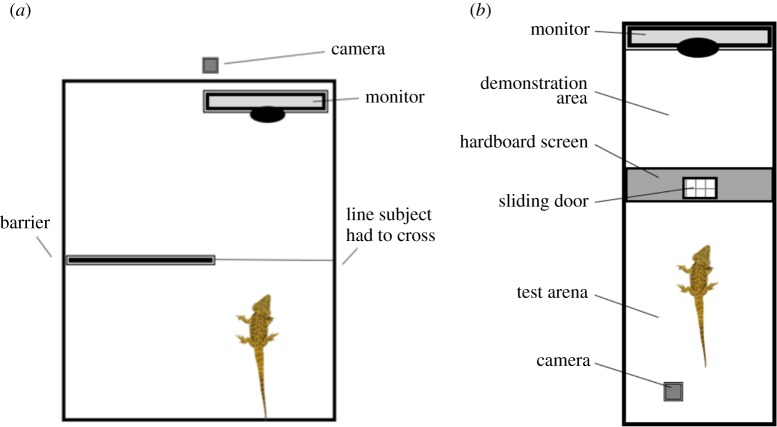
Plan of the experimental arena for (*a*) the gaze following experiment and (*b*) the social learning experiment. Not drawn to scale.

To assess gaze following, the lizards were placed facing towards a computer monitor within a familiar arena containing a barrier on one side (figure 1*a*). The experimental set-up consisted of a square arena measuring 73 × 73 cm with 19 cm high walls. A visual barrier (41 × 16 cm) was placed so that the lizard could see the screen but the barrier obstructed the view of the lizard to one side of the arena. A computer monitor was positioned in one end of the arena, this was used to present video stimuli to the observer animal (figure 1*a*). Bearded dragons have been shown previously to respond to videos of conspecifics [35].

Prior to the onset of the experiment all lizards were habituated to the arena. Each habituation trial lasted 10 minutes. During this time, the lizards had access to the entire arena including the barrier. Mealworms were placed inside the arena and the animals were considered habituated if they readily explored and ate all the food on two consecutive trials. All the lizards were habituated within two trials.

During the experimental trials, the observer animal was placed facing towards the screen. If the observer was not looking at the screen (classed as at least one eye facing towards the screen) or the lizard moved off before the video was played, then the trial was terminated and repeated on another day. Exactly 5 s after the bearded dragon had been placed in the arena, a video was presented. The video footage, described in more detail below, showed an unfamiliar female bearded dragon doing one of four things; looking up, looking to the side, looking to the side behind a barrier and looking straight ahead (the control). The gaze movements were recorded by presenting a favoured food in a specific position (above or to the side) relative to the demonstrator bearded dragon. The average length of the video clips was 2.33 ± 1.23 s and a different video was used for each trial. Once the clip was finished, the demonstrator bearded dragon remained on the screen and continued facing in the same direction. All trials were recorded on a video camera (Sony HDR-CX22OE) and were analysed using VLC media player.

#### 2.1.1. Look up into distant space

Each video showed a demonstrator lizard, positioned in the centre of the screen, looking up by moving its head upward with at least one eye looking upwards. An observer look-up was counted if the observer dragon looked up by either extending its head and neck upwards or by turning its head so one eye was directed upwards in the 5 s following the stimulus presentation.

#### 2.1.2. Look sideways into distant space

Each video showed a demonstrator lizard, positioned in the centre of the screen looking to the side by moving its head either to the left or the right (direction counterbalanced across animals) without tilting its head upward. The observer lizard was considered to be looking sideways if its head moved sideways in either direction in the 5 s following the stimulus presentation. Bearded dragons have binocular vision meaning that in order to follow the gaze of a conspecific the lizard could move its head to the side in either direction.

#### 2.1.3. Looking sideways behind the barrier

Each video showed a demonstrator lizard looking sideways behind a barrier. This meant that the observer lizard had to reposition itself in order to follow the gaze of the demonstrator lizard. The lizard was considered to have moved past the barrier if it moved so that its head was positioned so it could clearly follow the gaze of the demonstrator lizard or if it climbed over the barrier within the 1 min trial time. The video stimuli used in this condition presented identical information to those used in the *look sideways into distant space condition*; as such, the specific videos used for the two conditions were counterbalanced across subjects. The side was also counterbalanced (and the barrier was moved accordingly) across subjects.

#### 2.1.4. Control: stationary demonstrator

To control for the influence of the presence of a conspecific we included a condition in which the video showed a demonstrator lizard facing towards the observer but did not shift its gaze direction. When presented with the control condition, observers were expected to show fewer shifts in their gaze in the 5 s following stimulus presentation and move around the barrier less often than in the geometric gaze following condition.

Each lizard received one session of three trials a day repeated over four days. When an animal moved before the start of the video the trial was repeated the next day, hence it took five days to test all lizards. Each trial was separated by an inter-trial interval of at least 10 min, during which time the lizard was returned to its enclosure. Each animal received 12 trials in total and three trials per condition. The order of the trials was counterbalanced between animals and across sessions.

### 2.2. Data and statistical analysis

All trials were coded from video recordings. If the lizard responded within the appropriate time period (5 s after the start of the video for gaze following into the distance and 1 min for the moving around the barrier), it was coded as 1 and if it did not then it was coded as 0. These numbers were used as the dependent variable in a general linear model while incubation temperature and test condition were used as fixed factors as was the interaction between them. The individual was used as a random factor. We also used time to respond to gaze as a dependent variable in the same model to assess if incubation influenced the time it took the subject to respond to the demonstrator gaze. Ten per cent of the data was analysed by a second individual who was blind to condition and the inter-observer reliability was excellent (Cohen's *k* = 0.913, *p* = 0.001). All statistical analyses were carried out using Minitab (v. 17).

### 2.3. Social learning

To investigate social learning, we used a bi-directional control procedure [23] in which the lizards observed a video of an unfamiliar lizard opening a sliding door with its foot or with a sliding head movement to receive food behind it (figure 1*b*). After watching the video, the animals were given access to the sliding door and they had 5 min to open the door themselves. Each lizard was presented with 10 trials. The experimental arena (120 × 41.5 × 51 cm; length × width × height) was divided into two parts by the test apparatus (figure 1*b*); the test area (where the subjects were located) and the demonstration area (where a computer screen was located; figure 1*b*). The test apparatus was a wooden board (41.5 × 51 cm) with a horizontally sliding door with vertical bars in front of the hole (12 × 12 cm). The door could be opened to either the left or the right side. The sides of the arena were opaque and the floor was lined with newspaper. All testing was recorded with a digital camera (Panasonic HC-V100) on a tripod positioned above the arena.

All animals were habituated to the arena in a similar manner to the previous experiment. During the experiment the lizards received up to two trials per day, with a total of 10 trials per animal. At the onset of each trial the lizard was placed in the arena for 30 s. They then watched a demonstration video lasting 11 s in which they observed an unknown female demonstrator opening a horizontally sliding door to either the left or the right side, using a specific head movement (see [23] for full details). If the lizard moved away from the demonstration area prior to the start of the video, the lizard was placed in front of the screen before the video started. The observer animals saw either a demonstration in which the door was opened in a rightward direction or a leftward direction (a mirrored version of the stimulus). Each lizard was pseudo-randomly assigned either leftward or rightward opening demonstrations. This was counterbalanced across incubation condition.

After the video presentation, the observer lizard was then moved to the test area and placed behind a screen while the experimenter setup the trial (approx. 15 s). After the screen was raised the lizard had five minutes to open the sliding door to access a reward of a mealworm that was located behind the door. The trial was terminated when the lizard successfully opened the door and ate the reward or after five minutes had passed. Lizards were returned to their vivarium between trials.

The lizards were considered to have successfully opened the door when the door was moved enough, to either the right or left side, to create a visible gap (see electronic supplementary material, video S1 of a successful trial). The time taken before a successful opening was measured, as was the latency before attempting to open the door; latency ended when the subject first moved to make contact with the door. To investigate whether differences in motivation could account for any differences in performance between the two groups we assessed motivation by recording the sum of the amount of head and claw interactions with the door prior to its opening and measuring the latency to approach the door. A control condition was not included in this experiment as Kis *et al*. [23] showed that, over the course of 10 trials, bearded dragons were unable to complete this task without first observing a conspecific succeed.

### 2.4. Data and statistical analysis

For the door opening the data for the hot and the cold groups were compared using an independent *t*-test assuming unequal variances. A general linear model was used to see if there was a difference in the speed of social learning for the hot and the cold group. The time it took to open the door was used as a dependent variable, temperature was used as a fixed factor and the trial number was used as a covariant. Ten per cent of the videos were second coded and the correlation of results was excellent (*ρ*_13_ = 0.752, *p* = 0.003). All the statistical analysis was calculated on Minitab (v. 17).

## 3. Results

### 3.1. Gaze following

**Figure 2.**
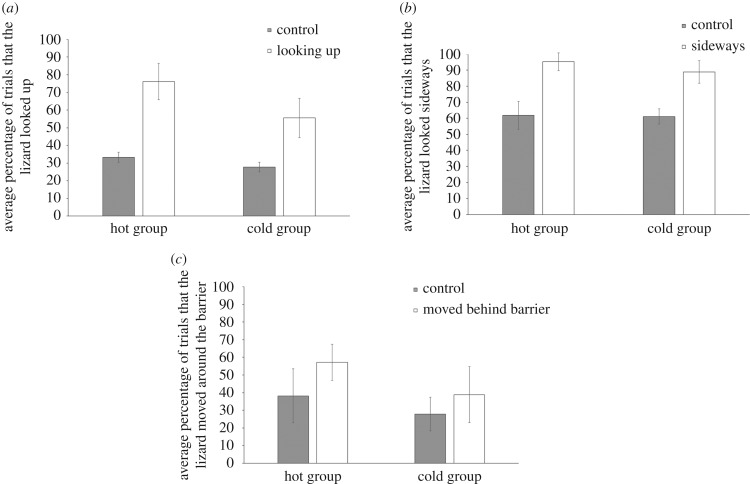
Mean percentage (±s.e.) of trials that the bearded dragons (*a*) looked up during the looking up condition compared to the control; (*b*) looked to the side during the looking sideways condition compared to the control; and (*c*) moved around the barrier in the geometric gaze following experiment.

Bearded dragons followed the gaze direction of the stimulus animal significantly more than they looked in that direction during the control trials when both looking upwards (*F*_1,23_ = 10.89, *p* = 0.003; figure 2*a*) or looking sideways (*F*_1,23_ = 21.49, *p* = 0.001; figure 2*b*). Egg incubation temperature did not influence their propensity to follow gaze into the distance (looking upwards: *F*_1,23_ = 1.44, *p* = 0.242; looking sideways: *F*_1,23_ = 0.29, *p* = 0.597). Lizards incubated at colder temperatures, however, were quicker to respond in general in the looking upwards but not the looking sideways condition (looking upwards: *F*_1,24_ = 5.59, *p* = 0.027; looking sideways: *F*_1,34_ = 0.53, *p* = 0.975; electronic supplementary material, figures S1 and S2). There was no evidence of geometric gaze following in the bearded dragons; individuals were equally as likely to move around the barrier on a control trial as they were when they observed the stimulus animal looking behind the barrier (*F*_1,23_ = 1.44, *p* = 0.242; figure 2*c*); this was also unaffected by incubation temperature (*F*_1,23_ = 1.24, *p* = 0.277).

### 3.2. Social learning

**Figure 3.**
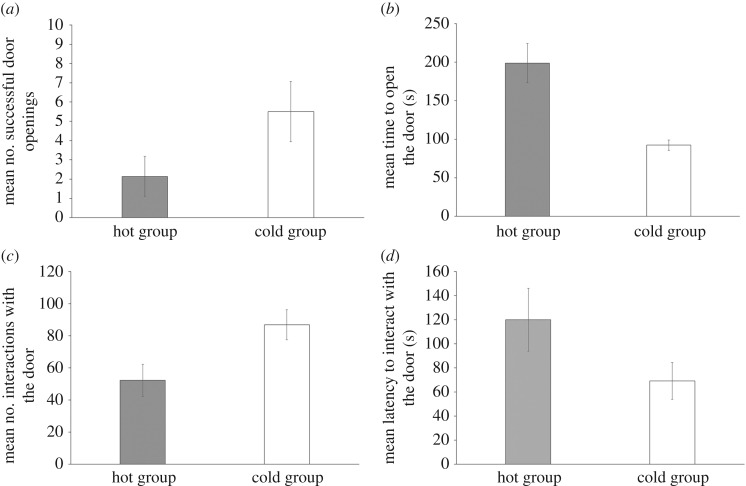
(*a*) Mean (±s.e.) number of trials that the hot-incubated and cold-incubated groups of lizards opened the door; (*b*) the mean (±s.e.) time taken per trial to open the door; (*c*) the mean (±s.e.) number of interactions with the door; and (*d*) the mean (±s.e.) latency in seconds for the lizard to touch the door.

**Figure 4.**
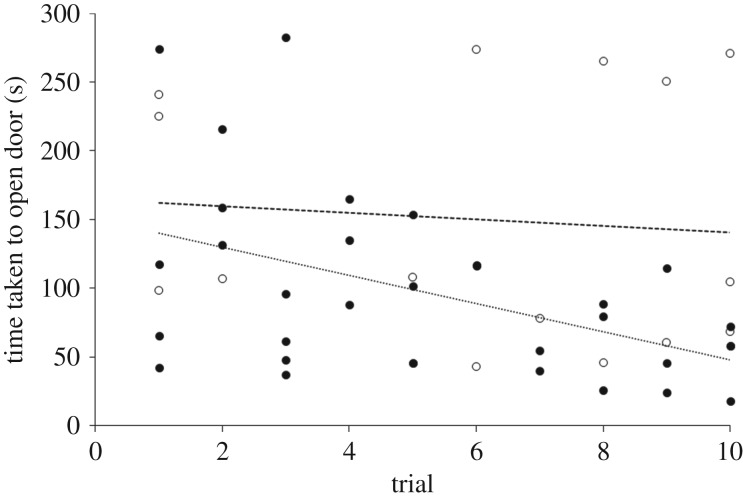
The relationships between the time taken by bearded dragons to open the door during consecutive trials for cold-incubated (•, solid line) and hot-incubated (○, dashed line) bearded dragons.

Although the ‘cold group’ opened the door more times than the ‘hot group’ (figure 3*a*) this difference was not significant (*t*_8_ = −1.79, *p* = 0.111). However, over the course of 10 trials, the cold group completed the task significantly quicker than the hot group (figure 4; trial: *F*_1,45_ = 4.95, *p* = 0.03; temperature: *F*_1,45_ = 7.05, d.f. = 1, *p* = 0.011) and spent significantly less time (92.3 ± 7.2 s) than the ‘hot group’ (199.0 ± 30.3 s) attempting to open the door (*t*_4_ = 3.43, *p* = 0.027: figure 3*b*). The number of times the animals touched the door (figure 3*c*), significantly differed between the ‘hot’ and ‘cold group’ (*t*_10_ = −2.52, *p* = 0.031) but the latency to touch the door was not significantly different between the hot and cold groups (*t*_9_ = −1.68, *p* = 0.127; figure 3*d*).

## 4. Discussion

Our findings reveal that egg incubation environment impacts upon some aspects of social cognition in adult bearded dragons. We found that the lizards were able to follow the gaze of a conspecific into distant space, but that both incubation groups performed similarly in this task and neither group were able to follow the gaze of a conspecific around a barrier. By contrast, there was an effect of incubation temperature on social learning, with the cold-incubated animals performing significantly faster than those that were incubated at a warmer temperature over time.

Gaze following into the distance is thought to be controlled by an innate orienting response, which can be modulated by experience [13,18]. This is generally considered to be a relatively simple mechanism and, as such, it may not be surprising that no differences were observed in performance of this task. However, the results did suggest that lizards from the cooler incubation group looked up more rapidly than the hotter incubated animals. By contrast, geometric gaze following is considered cognitively complex because, at a minimum, it requires either learning about how barriers impair vision [14] and using that information appropriately or, potentially, forming a mental representation of the demonstrator's visual perspective [13]. Here, at least under these conditions, the bearded dragons were unable to use this information irrespective of their incubation environment. It is not clear whether this lack of effect represents a true lack of ability; however, the lizards moved around the barrier in 40% of all trials, suggesting that neophobia was not the reason for the failure in this task.

We found a significant influence of egg incubation temperature on social learning. Over the course of the experiment, the cold-incubated animals opened the door significantly faster than the hot-incubated animals. The nature of this difference supports the idea that the contrast observed between the groups in this experiment may be the result of differences in associative learning abilities caused by incubation environment. It, therefore, provides further evidence for the idea that associative learning mechanisms may underpin social learning. This is supported by research in a number of areas. The medial cortex is thought to play a central role in reptile learning [36] and recent work has revealed that incubation temperature positively influences the density of neurones found in this region of the brain in hatchling Eastern three-lined skinks [6], though it remains unclear whether other brain areas are also impacted by the manipulation, and to what extent this observation applies to other species. Further, previous research has observed differences in associative learning abilities as a result of incubation environment in other species [6–9,32]. Taken together, the results add to the idea that associative processes play a crucial role in social learning abilities.

There was a difference in the number of interactions with the door between the conditions, with the cold-incubated animals interacting with the door more than the hot-incubated animals. This suggests that there may be a difference in motivation between the two groups. However, there was no difference in willingness to approach the door. This, in combination with recent work with the same animals, which revealed that there was no difference in food motivation between the two groups [37], makes it unlikely that the differences observed were the result of this, but were rather the result of differences in social learning ability.

All previous research in this area has used very young animals [6–9,32] and it was unclear whether the observed differences in cognitive ability persisted during ontogeny. Our previous work revealed that incubation environment impacted upon the development of behavioural traits but that these did not differ when the animals were adults [33]. Although most studies deal with short-term effects of incubation conditions [5], there is evidence that long-term (over a year) survival in snapping turtles (*Chelydra serpentina*) is affected by incubation temperature [38], which implies an effect on individual fitness. Although the effect of egg incubation temperature on social cognition in young bearded dragons is unknown, the results are the first to reveal that incubation environment can influence the cognitive ability of adult reptiles. The results come from captive animals; however, they suggest that if this behaviour was seen under natural conditions it is likely to have profound impact upon individual fitness.

The mechanisms that underlie temperature-dependent differences in the phenotypes of oviparous reptiles remain poorly understood [5,36]. One intriguing idea suggests that incubation environment may ‘select’ for traits that are adaptive to the specific environment into which the animal is born [33]. Therefore, a cooler environment may produce animals that are better adapted to survival in that temperature profile and *vice versa*. Further research is required to test these ideas. As such, it is possible that variation in the sensitivity of oviparous reptiles to external environmental factors may provide a behavioural buffer that allows individuals to better cope with heterogeneous and changing environments.

## Supplementary Material

Figure S1 & S2

## Supplementary Material

Data

## Data accessibility

We provide supporting data in the supplementary information.
