# Supplementary material for: Incubation environment impacts the social cognition of adult lizards
Source: R Soc Open Sci. 2017 Nov 22;4(11):170742. doi: 10.1098/rsos.170742 (PMC5717640; doi:10.1098/rsos.170742)
Supplement: Figure S1 & S2 [file rsos170742supp1.docx]

**Incubation environment impacts the social cognition of adult lizards**

Harry Siviter^1,2^, D. Charles Deeming^1^, M. F. T. van Giezen^1,3^ & Anna Wilkinson^1,4^

^1^School of Life Sciences, University of Lincoln, Lincoln, LN6 7DL, UK; ^2^ School of Biological Sciences, Royal Holloway University of London, Egham, UK; ^3^Faculty of Veterinary Medicine, Utrecht University, The Netherlands; ^4^Wildlife Research Center, Kyoto University, 2-24 Tanaka-Sekiden-cho, Sakyo, Kyoto, 606-8203, Japan

**Supplementary Figures**

**
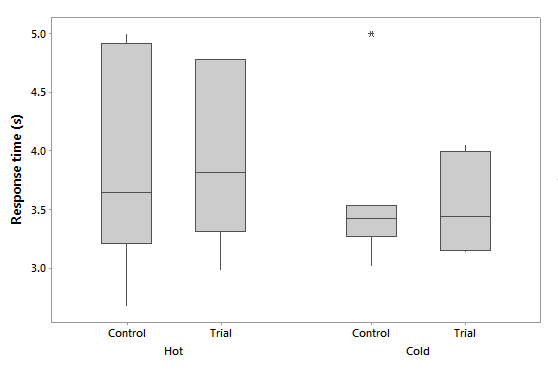
**

**Figure S1: Looking response time for the looking upwards condition.**

**
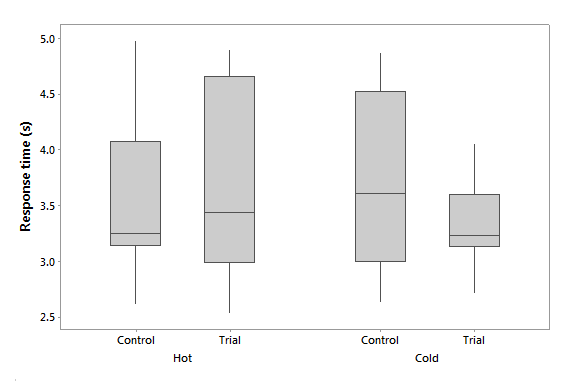
**

**Figure S2. Looking response time for the looking sideways condition.**
